# Effects of selective serotonin reuptake inhibitor (SSRI) use on cardiometabolic health and risk in young healthy individuals: A preliminary matched pairs study

**DOI:** 10.14814/phy2.70285

**Published:** 2025-04-26

**Authors:** Elena S. Shostak, Jillian M. Lang, William K. Quinn, Valerie D. Chervinskaya, Elisa Fioraso, Everett Smith, Christopher J. Kotarsky, Justin A. DeBlauw, Jennifer L. Lloyd, Stephen J. Ives

**Affiliations:** ^1^ Health and Human Physiological Sciences Skidmore College Saratoga Springs New York USA; ^2^ Biomedicine and Movement Science University of Verona Verona Italy; ^3^ Oxford College of Emory University Atlanta Georgia USA; ^4^ Rehabilitation, Exercise, and Nutrition Sciences University of Cincinnati Cincinnati Ohio USA; ^5^ Kinesiology and Outdoor Recreation Southern Utah University Cedar City Utah USA; ^6^ Optum Home Infusion Services Eden Prairie Minnesota USA

**Keywords:** arterial stiffness, body composition, cardiac autonomic nervous system activity, central blood pressure, mental health, microvascular function

## Abstract

Selective serotonin reuptake inhibitors (SSRIs) are a class of drugs that are frequently used to treat psychological disorders, but the comprehensive effects on cardiometabolic health in young healthy populations are not well described. Healthy men and women using SSRIs and sex‐, age‐, height‐, and weight‐matched controls were assessed. Anthropometrics, blood pressure (BP), arterial stiffness (AS), heart rate variability (HRV), near‐infrared vascular occlusion test (NIRS‐VOT), and blood lipid profile were assessed, with subsequent Framingham CVD risk score calculation. There were no significant differences in central or peripheral BP or AS due to SSRI use (all, *p* > 0.39, Cohen's d < 0.48). HRV was not different between groups (all, *p* > 0.43, Cohen's d < 0.44), except for HFpeak, which was lower in SSRI users (0.20 ± 0.05 vs. 0.28 ± 0.06 Hz, *p* = 0.04, Cohen's d = 1.26). There were no significant differences in blood lipids (all, *p* > 0.22, Cohen's d < 0.09) or Framingham risk scores (both, *p* > 0.68, Cohen's d < 0.14) between groups. There were no significant differences in microvascular reactivity between groups, however, reperfusion slope was lower in SSRI users (1.2 ± 0.9 vs. 2.0 ± 1.1%/s, *p* = 0.10, Cohen's d = 0.93). SSRIs do not appear to have any significant effects on blood pressure, heart rate variability, arterial stiffness, or lipid profile in young healthy individuals but may influence microvascular function.

## 1 INTRODUCTION

Serotonin is a neurotransmitter that, when dysregulated, has been linked to mental health disorders, specifically depression and anxiety (Peixoto et al., 2019). Depression, which is a mental health condition involving a persistent low mood and loss of interest in activities, is a significant contributor to mortality, morbidity, and disability in the United States (U.S.) (Lee et al., 2023). In 2020, nearly 20% of U.S. adults reported ever having been diagnosed with depression (Lee et al., 2023). Generalized Anxiety Disorder (GAD), characterized by excessive worrying along with restlessness and other physical symptoms, is prevalent in the U.S. as well, with 16% of U.S. adults experiencing GAD symptoms in 2019 (Moron et al., 2023). The relationship between depression, GAD, and serotonin dysregulation has been thoroughly researched (Peixoto et al., 2019). Individuals with depression and/or anxiety are often more sedentary, less adherent to medications, more likely to smoke and abuse alcohol, and less likely to be able to make healthy lifestyle changes (Peixoto et al., 2019), which are habits that can contribute to hypertension (Arnett et al., 2019). It has been demonstrated that serotonin dysregulation and hypertension are related; therefore, it follows that depression and hypertension have been linked in epidemiological studies (Peixoto et al., 2019; Watts et al., 2012). However, evidence is scarce on the impacts of SSRI use on central blood pressure, a novel and independent risk factor for cardiovascular disease that is likely superior to brachial blood pressure (Roman et al., 2007), which warrants more research in this area.

Numerous drugs are prescribed to treat anxiety and depression. Selective serotonin reuptake inhibitors (SSRIs) are a class of antidepressants used to manage psychological disorders through increasing serotonin bioavailability (Calvi et al., 2021; Ungvari et al., 2019). SSRIs function by blocking presynaptic reuptake, mediated by serotonin transporters, thus promoting and prolonging serotonergic neurotransmission (Calvi et al., 2021; Ungvari et al., 2019). The usage of SSRIs has steadily increased over the past 20 years, likely due to increased diagnoses of depression and anxiety (Murphy et al., 2021). The increase in SSRI prescription and use calls for more research regarding its effects, especially in vulnerable adults or those with existing diseases, but also in healthy individuals. Research on the relationship between SSRIs and cardiovascular health remains contentious. Antidepressants alter multiple neurotransmitter systems, indirectly or directly, affecting blood pressure (BP) regulation (Calvi et al., 2021; Ungvari et al., 2019). SSRIs have demonstrated significant vascular effects in unhealthy and elderly populations that include myogenic constriction of blood vessels, implicating orthostatic hypotension, and mild bradycardia (Roman et al., 2007; Zhang et al., 2023). Venlafaxine, for example, is an SSRI that may provoke dose‐dependent BP elevation by 7 mmHg with doses of 300 mg or more (Kıvrak et al., 2014). However, a meta‐analysis of six studies on cardiovascular disease (CVD) and depression demonstrates that there are no significant effects of SSRIs on BP in hypertensive patients (Zhang et al., 2023). However, there is very little research on the effects of SSRIs on cardiometabolic health more broadly using a comprehensive assessment in young and otherwise healthy populations, which is surprising given the rates at which SSRIs are used for treating anxiety/depression in this population.

Therefore, the purpose of this study was, using a matched pairs design, to examine the effect of SSRI use on cardiometabolic health, specifically blood pressure, vascular stiffness, microvascular function, heart rate variability, and blood lipid profiles in young healthy individuals. It was hypothesized that SSRI would negatively impact blood pressure, either through altered estimated autonomic nervous system activity (heart rate variability) or altered vascular resistance (vascular function). Further, we expected SSRI use to be associated with poorer cardiometabolic health and cardiovascular disease risk in a relatively young population, who are, otherwise healthy, even after matching for sex, age, height, weight, and BMI.

## 2 METHODS

### 2.1 Subject and general procedures

Nonsmoking healthy men and women were recruited via email from Skidmore College and the surrounding community. In a matched pairs design, using a one‐tail approach, a large effect size, and alpha of 0.05 to achieve a minimum power of 0.8, a total of 12 participants would be needed. Once SSRI users were identified, we aimed to find age‐, sex‐, height‐, and weight‐matched controls. Participants were between the ages of 18 and 41 years and were screened for uncontrolled chronic diseases (e.g., cardiovascular, metabolic, and pulmonary) as well as risk factors for cardiovascular disease. Exclusion criteria were current or recent (<3 months prior) usage of oral antibiotics or irritable bowel syndrome, or gut/bowel maladies diagnosis (Mohr et al., 2022), two or more CVD risk factors (Costa et al., 2018), cancer or cancer treatment, and treatment of hypothyroid with levothyroxine, as well as those with uncontrolled cardiovascular, metabolic, or pulmonary diseases. Participants who recently (<8 weeks) donated blood were excluded. Women who were pregnant, breastfeeding, attempting to conceive, or amenorrheic were also excluded from the study. Those with severe illness, compromised/suppressed immune systems, and/or eating disorders and food allergies were excluded. Participants were asked to come into the laboratory under standardized conditions; namely, they were asked to continue any currently prescribed medications but avoid any supplements (e.g., vitamins, nutraceuticals [herbs, extracts, etc.], weight loss pills, etc.) before the study. Participants were also asked to avoid strenuous exercise for 24 h and caffeine/alcohol intake for 12 h preceding the study visit. Participants provided written informed consent prior to participation. The study was approved by the Institutional Review Board at Skidmore College (IRB#2209‐1044) and was done according to the most recent revisions to the Declaration of Helsinki. This study was part of a larger trial registered with clinicaltrials.gov (NCT06544915).

### 2.2 Procedures

The current study used a single‐visit, cross‐sectional, matched‐pairs design. During the consenting process, participants documented their use of prescription medications and were then grouped into those who self‐reported the use of prescribed SSRIs and those who did not. Once the SSRI users were identified, controls were then identified to match based on sex, age, height, and weight. Data were collected and analyzed by investigators who were blinded to which group participants were in.

### 2.3 Measurements

#### 2.3.1 Anthropometrics

Upon arrival at the laboratory, the participants' height and body composition (total body, fat, and fat‐free mass and body water) were assessed using a stadiometer (Seca, Mt. Pleasant, SC, USA) and body composition scale (RD‐545, Tanita, Arlington Heights, IL, USA), respectively. Bioelectric impedance analysis (BIA) assessed conductivity, providing estimates of body water content; therefore, distinguishing fat mass and fat‐free mass. The Tanita BIA has been documented to be a reliable and valid approach (Vasold et al., 2019), and we have published typical in‐house testing data documenting an average within‐day coefficient of variation of 0.4% across three trials (DeBlauw et al., 2023).

#### 2.3.2 Blood pressure and vascular stiffness

Using an oscillometric cuff (Sphygmocor Xcel, Atcor, Naperville, IL, USA) peripheral blood pressures were recorded, and with a subsequent generalized transfer function, central blood pressures were estimated (Shoji et al., 2017), which provides independent prognostic value (Roman et al., 2007). Pulse wave analysis (PWA) was conducted using the derived central pressure waveforms, which calculated augmentation pressure (AP), augmentation index (AIx), and AIx normalized to 75 beats per minute HR (AIx@75).

#### 2.3.3 Heart rate variability (HRV)

Participants were given a HR monitor (H10, Polar, Lake Success, NY, USA), which was strapped around the chest, and then positioned supine and allowed to quietly rest for 10 min before any measurements were made. To understand the potential for SSRI use to alter autonomic nervous system outflow, HR and HRV were measured using the HR monitor. The monitor transmitted data to a mobile device via Bluetooth with an automated software application (Elite HRV, v. 5.5.8, Gloucester, MA, USA) (Egan‐Shuttler et al., 2020). The application performed artifact correction and provided time‐domain and frequency‐domain HRV metrics, as used previously (Matias et al., 2023; Shostak et al., 2024; Zaleski, Gyampo, et al., 2023. A 5‐min recording was captured under spontaneous breathing conditions.

#### 2.3.4 Near‐infrared spectroscopy vascular occlusion test (NIRS‐VOT)

The NIRS‐VOT was conducted as described previously (Blum et al., 2024; Greaves et al., 2024; Zaleski, Matias, et al., 2023) to measure microvascular reactivity. After cleaning the right medial forearm with an alcohol swab and shaving any hairs present, a NIRS device (MOXY, Fortiori Design LLC, Hutchinson, MN, USA) was attached to the right medial forearm by wrapping an opaque bandage around the device. An occlusion cuff was placed proximal to the NIRS device on the bicep of the same arm and set to 220 mmHg (E20, Hokanson, Bellevue, WA, USA). After 10 min of rest, baseline tissue oxygen saturation was captured for 1 min. The cuff was then occluded for 5 min to capture the desaturation slope after cuff inflation (slope 1) which was quantified 30–150 s after cuff inflation. The cuff was then released, and an additional 1 min of data was collected. The reoxygenation slope (slope 2) was captured in the first 10 s after the cuff was deflated.

#### 2.3.5 Blood glucose and lipid profile

Using the standard capillary fingerstick technique, blood samples were collected using a pressure‐activated lancet in the fingertip of the participant's preferred hand. About 40 μL of blood was used for lipid (define out variables here) and glucose analysis in a point‐of‐care testing device (Cholestech LDX Analyzer, Abbott, Lake Forest, IL, USA), which has been validated against standard clinical testing (Carey et al., 2006). The unit was checked for optical properties each day and tested against low‐ and high‐level controls monthly. The Cholestech provides measurements of high‐ and low‐density lipoprotein cholesterol (HDL and LDL), total cholesterol, non‐HDL cholesterol, total cholesterol and HDL ratio (TC/HDL), triglycerides, and glucose. We have previously documented an in‐house within‐day coefficient of variation of ≤2% on the blood glucose and lipid profile (DeBlauw et al., 2023).

#### 2.3.6 Framingham CVD risk scores

The Framingham Heart Study CVD risk factor calculations were used to determine the 10‐year risk of CVD and heart age. The risk calculator derives from the 1948 longitudinal study on healthy residents of Framingham, Massachusetts, who were observed over 20 years. Calculations are made based on sex, age, systolic blood pressure (SBP), treatment for hypertension, smoking status, diabetes status, high‐density lipoprotein cholesterol (HDL), and total cholesterol levels (D'agostino et al., 2008).

### 2.4 Statistical analysis

All statistical analyses were carried out using open‐source software (JASP, v 0.19, Amsterdam, Netherlands). Students *t*‐tests were used to determine any significant differences in body composition, BP, HRV, blood glucose and lipid profiles, and microvascular reactivity between participants taking SSRIs and those without medications. The Shapiro–Wilk test for normality was used to evaluate whether the data was normally distributed, and Levene's test for equality of variance was used to determine if assumptions were met. If not, a nonparametric alternative, Welch's test, was used. Cohen's d was used to determine the effect size between the two groups, with 0.2, 0.5, and 0.8 indicating small, medium, and large effects, respectively. Alpha was set at 0.05. Data were presented as mean ± standard deviation unless specified otherwise.

## 3 RESULTS

### 3.1 Participant characteristics

This study included 14 healthy male and female participants aged 18–41 years. By design, no significant differences between groups were observed in age, height, weight, and BMI (all, *p* > 0.05, Table 1). Although body composition was also not different (*p* = 0.116) there was a noticeable trend for the SSRI group to have higher % body fat with a large effect size (d = 0.9). Participants reported no other medications aside from hormonal contraceptives in female participants.

**TABLE 1:** Participant characteristics in SSRI users and matched nonusers.

Condition	SSRI	Matched nonusers	*p*	Cohen's d
Sex F/M	5/2	5/2	–	–
Age (years)	26 ± 10	25 ± 9	0.864	−0.09
Height (cm)	165.2 ± 10.5	169.7 ± 9	0.407	0.46
Weight (kg)	72.6 ± 15.7	73 ± 21	0.967	0.02
BMI (kg/m^2^)	26.6 ± 4.5	25.1 ± 4.7	0.547	−0.33
Body fat (%)	30.4 ± 6.9	24.6 ± 5.7	0.116	−0.90

*Note*: Data are mean ± SD.

Abbreviations: BMI, body mass index; F, female; M, male.

### 3.2 Blood pressure and vascular stiffness

**FIGURE 1:**
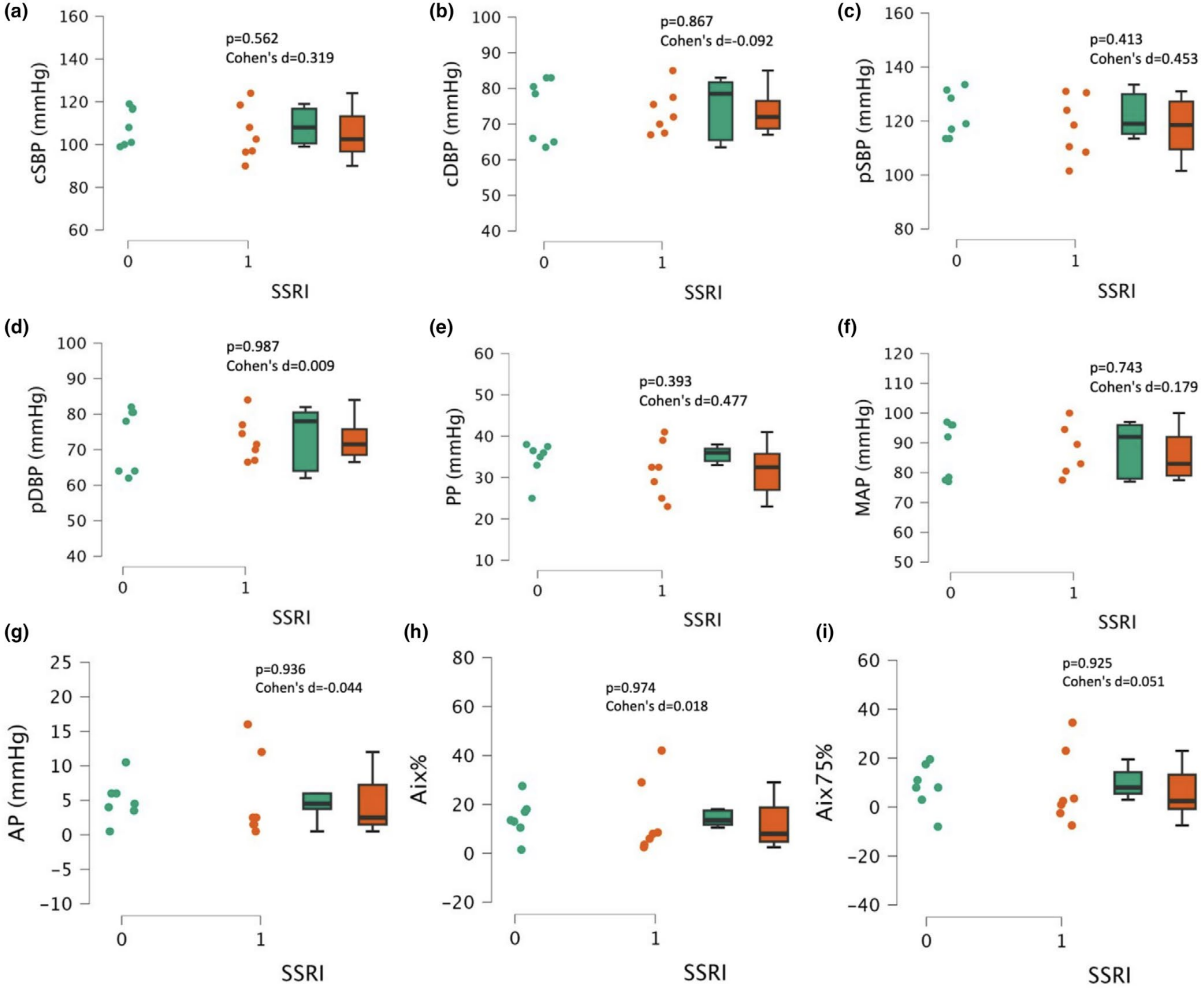
Blood pressure and vascular stiffness measurements in SSRI users (1, *n* = 7) and matched nonusers (0, *n* = 7). Central systolic blood pressure (cSBP, Panel a), central diastolic blood pressure (cDBP, Panel b), peripheral systolic blood pressure (pSBP, Panel c), peripheral diastolic blood pressure (pDBP, Panel d), pulse pressure (PP, Panel e), mean arterial pressure (MAP, Panel f), augmentation pressure (AP, Panel g), augmentation index (Aix, Panel h), and augmentation index 75% (Aix75%, Panel i). Data are presented as individual plots and as box and whiskers (median and IQR).

There were no significant differences in central systolic and diastolic blood pressure (cSBP and cDBP) between participants taking SSRIs and those not (*p* > 0.562, Cohen's d < 0.319, Figure 1a,b). There were no significant differences in peripheral systolic and diastolic blood pressure (pSBP and pDBP) between participants taking SSRIs and those not (*p* > 0.413, Cohen's d < 0.453, Figure 1c,d). There were no significant differences in measures of vascular stiffness (PP, MAP, AP, AIx%, AIx@75) between participants taking SSRIs and those not (all, *p* > 0.39, Cohen's d < 0.477, Figure 1e–i).

### 3.3 Heart rate variability

**FIGURE 2:**
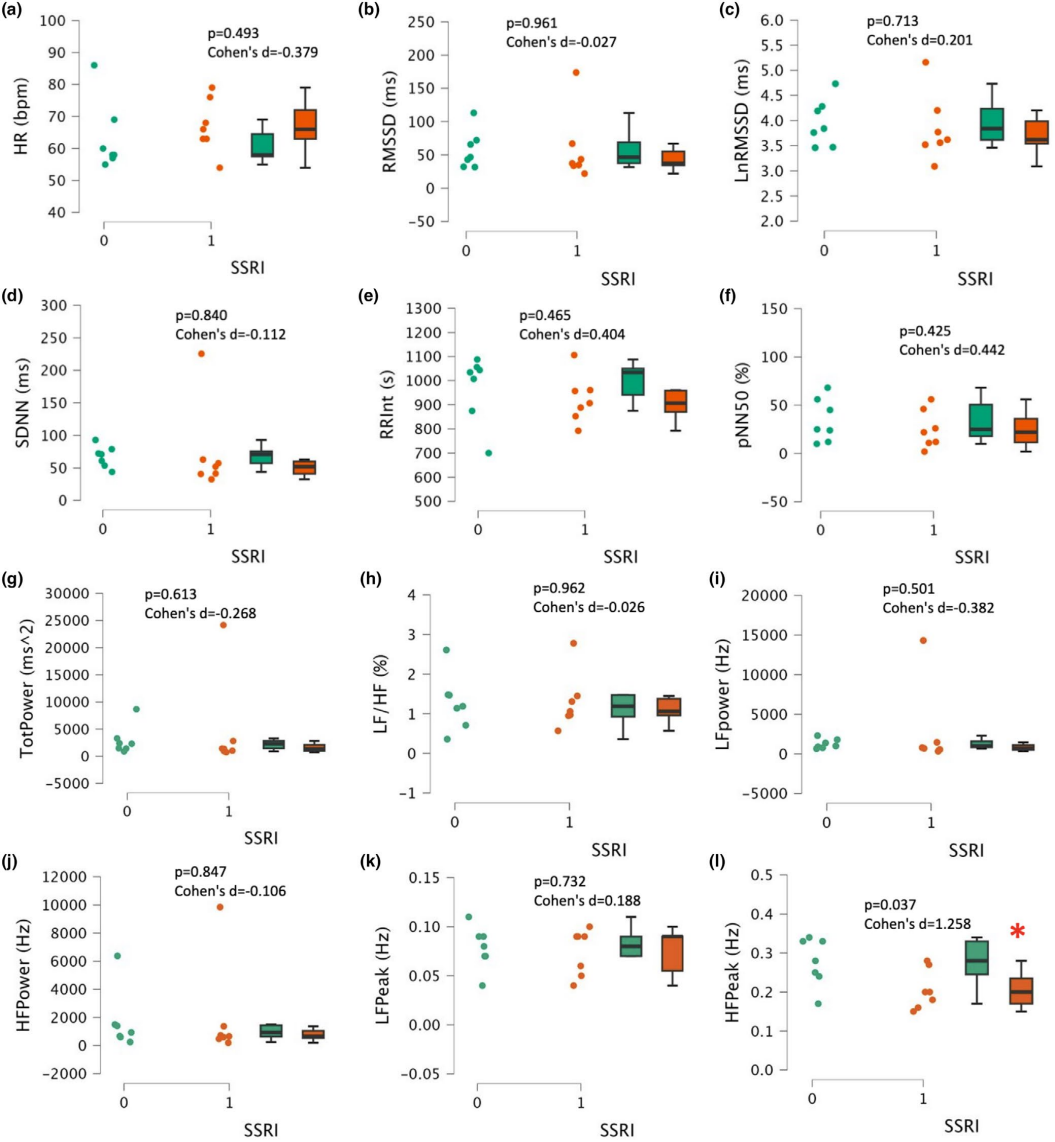
Heart rate variability (HRV) in SSRI users (1, *n* = 7) and matched nonusers (0, *n* = 7). (a) Heart rate (HR), (b) root mean square of successive differences (RMSSD), (c) natural log RMSSD (LnRMSSD), (d) standard deviation of normal‐to‐normal R‐R intervals (SDNN), (e) R‐R interval (RRInt), (f) percent of successive N‐N (R‐R) intervals >50 ms (pNN50), (g) total power (TotPower), (h) low frequency (LF) and high‐frequency (HF) ratio (LF/HF), (i) LF power, (j) HF power, (k) LF peak, (l) HF peak. Data are presented as individual plots and as box and whiskers (median and IQR). *p < 0.05 SSRI users versus age, sex‐matched controls.

There was not a significant difference in HR between the groups (*p* = 0.492, Cohen's d = −0.397, Figure 2a). There were no significant differences in HRV measurements between the groups (all, *p* > 0.425, Cohen's d < −0.382, Figure 2b–k); however, the HF peak was significantly larger in participants without medication at 0.277 ± 0.06 Hz compared to 0.206 ± 0.05 Hz (*p* = 0.037, Cohen's d = 1.258, Figure 2l).

### 3.4 Lipid profile and Framingham CVD risk scores

**FIGURE 3:**
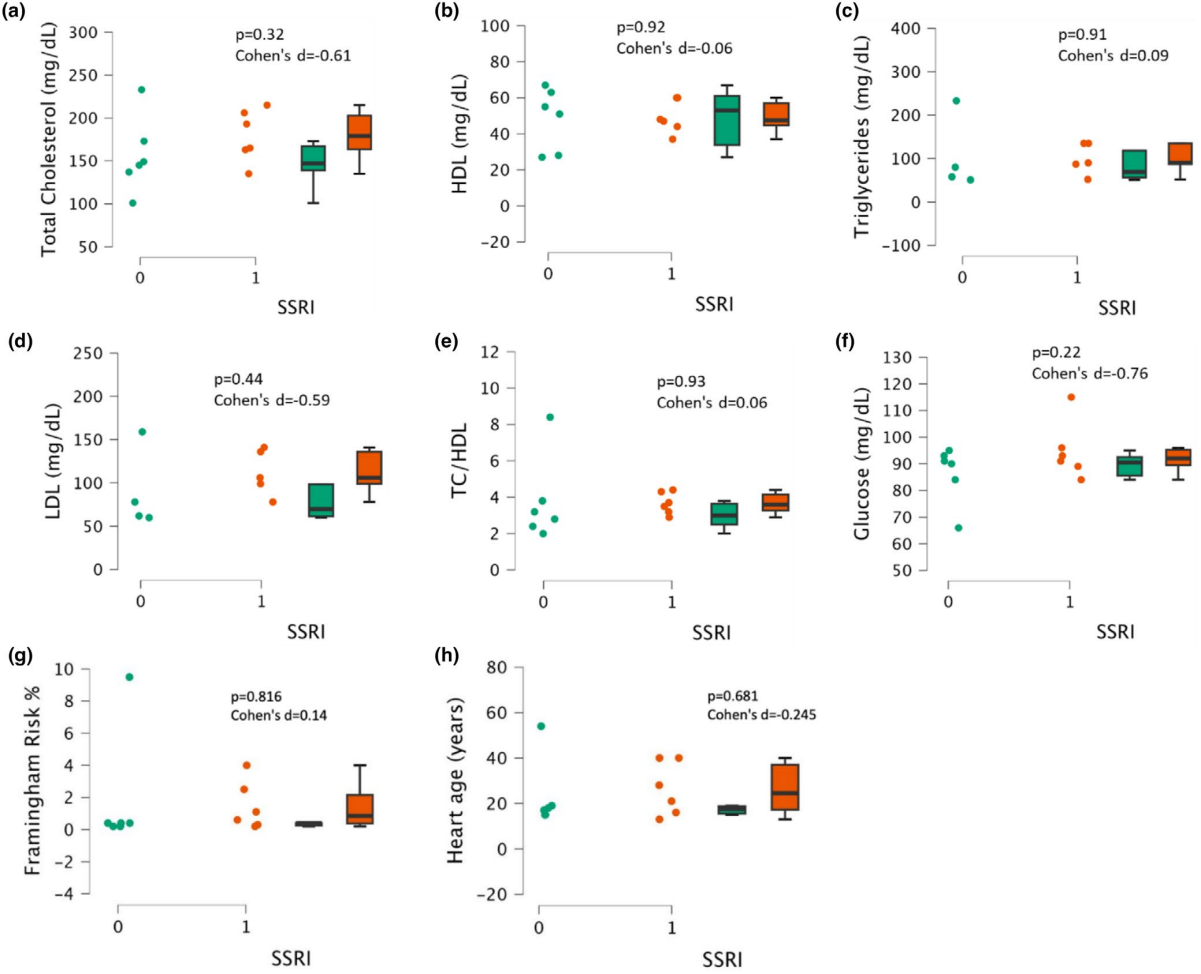
Blood lipid panel measures and Framingham CVD Risk scores in SSRI users (1, *n* = 7) and matched nonusers (0, *n* = 7). Total cholesterol (Panel a), high‐density lipoprotein (HDL, Panel b), triglycerides (Panel c), low‐density lipoprotein (LDL, Panel d), total cholesterol and high‐density lipoprotein ratio (TC/HDL, Panel e), glucose (Panel f), Framingham CVD risk score (Panel g) and heart age (Panel h). Data are presented as individual plots and as box and whiskers (median and IQR).

There were no significant differences in total cholesterol, HDL and LDL cholesterol, triglycerides, non‐HDL cholesterol, TC/HDL, and glucose between participants taking SSRIs and participants without medication (all, *p* ≥ 0.22, Cohen's d ≤ 0.09 Figure 3a–f). There was no significant difference in Framingham CVD risk scores between conditions, with SSRI users at an average of 1.45 ± 1.5% and nonusers at 1.85 ± 3.7% (*p* = 0.82, Cohen's d = 0.14, Figure 3g). There was no significant difference in heart age in years between conditions, with SSRI users at an average age of 26 ± 12 years and nonusers at 23 ± 15 years (*p* = 0.68, Cohen's d = −0.25, Figure 3h).

### 3.5 Microvascular function

**FIGURE 4:**
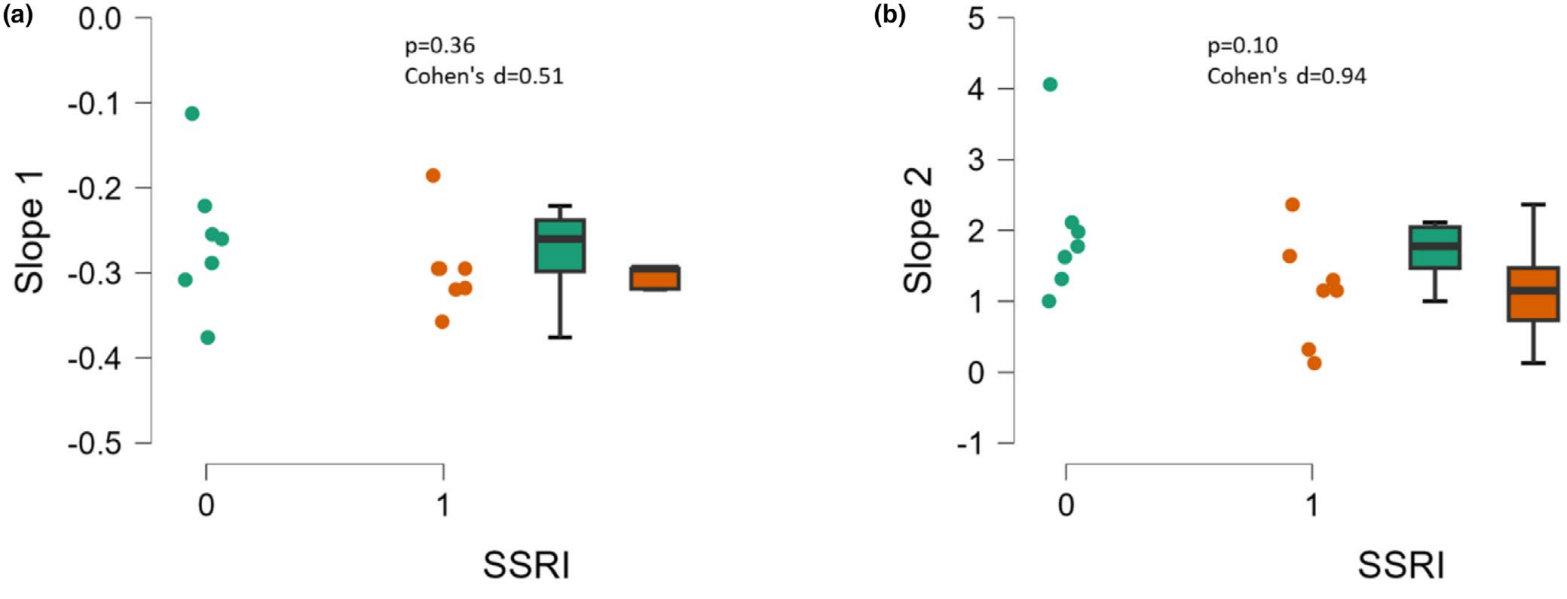
Muscle oxygen (SmO_2_) desaturation and reperfusion slopes from the near‐infrared vascular occlusion test (NIRS‐VOT) in SSRI users (1, *n* = 7) and matched nonusers (0, *n* = 7). SmO_2_ desaturation (Slope 1, Panel a) and SmO_2_ reperfusion (Slope 2, Panel b). Data are presented as individual plots and as box and whiskers (median and IQR).

There were no significant differences in indicators of microvascular health, assessed via slope 1 (*p* = 0.36, Cohen's d = 0.51) and slope 2 (*p* = 0.10, Cohen's d = 0.94) derived from the NIRS VOT, among participants taking SSRIs and those not (Figure 4a,b, respectively). However, slope 2 saw a large effect size of 0.94, with the average slope 2 of 1.98 ± 0.99%/s for non‐SSRI users and 1.15 ± 0.76%/s for SSRI users.

## 4 DISCUSSION

The purpose of this study was to examine the effects of SSRI use on cardiometabolic health, specifically central and peripheral blood pressure, vascular stiffness, microvascular function, heart rate variability, and blood and lipid profiles in young, healthy individuals. It was hypothesized that SSRI use would negatively affect cardiometabolic health in young, healthy populations. After matched‐pair analyses were conducted, no significant differences were found in body composition, BP, HR, HRV, and blood glucose and lipid profiles between participants currently using SSRIs and participants who were not using SSRIs. However, the HRV metric HF peak was lower with SSRI use, suggesting lower parasympathetic nervous system activity and microvascular function that tended to be ~40% lower with SSRI use. Utilizing traditional risk factors to calculate Framingham risk scores, there were no significant differences between those using SSRIs and controls. The present investigation offers supporting evidence that SSRI use in relatively young healthy individuals does not appear to cause any significant adverse effects on traditional cardiovascular risk factors, although investigation of emerging preclinical risk factors (i.e., body composition and vascular function) may be useful in assessing risk in future studies exploring longer‐term use of SSRIs or in future trials.

### 4.1 Effects of SSRIs on body composition

To determine the potential impact of SSRI use on body weight, Cockerill et al. reviewed medical records of overweight adolescents medicated with SSRIs and non‐medicated to determine potential differences in body mass index (BMI) (Cockerill et al., 2014). After analyzing 435 patients, researchers found that SSRIs significantly increased weight in overweight adolescents, while non‐medicated patients saw no change in weight (Cockerill et al., 2014). A 2020 study executed by Ostrowska et al. observed the effects of SSRIs on body composition of men and women separately (Ostrowska et al., 2020). After analyzing 107 patients treated with antidepressants and 104 healthy individuals, researchers found a significantly higher BMI in men taking SSRIs compared to healthy men, while the average BMIs of women in each condition were similar (Ostrowska et al., 2020). In the present study, by design, BMI was not significantly different between the two groups. However, despite similar BMI, body fat percentage tended to be 24% higher in the SSRI group (large effect size, d = 0.9), suggesting that SSRI use might be associated with increased adiposity, but this warrants further study to ascertain whether this effect is spurious or casual in nature.

### 4.2 Effects of SSRIs on blood pressure and arterial stiffness

Previous research on the effects of antidepressants on CVD risk factors remains heterogeneous, especially for SSRI drugs. In a randomized double‐blind placebo‐controlled trial performed by Glassman et al., 369 patients with myocardial infarction or unstable angina were randomly assigned either the SSRI drug sertraline or a placebo for 24 weeks of treatment (Glassman et al., 2002). The primary variable focused on was left ventricular ejection fraction (LVEF); however, BP was also measured (Glassman et al., 2002). There were no statistically significant differences in LVEF or BP between patients receiving sertraline and placebo (Glassman et al., 2002). Incidences of cardiac events were not significantly different between groups; however, interestingly, it was numerically lower among patients who were receiving the SSRI (Glassman et al., 2002). In a 12‐week placebo‐controlled study by Wilens et al., 107 children and 80 adolescents with obsessive‐compulsive disorder (OCD) were treated with doses of sertraline less than or equal to 200 mg per day (Wilens et al., 1999). Researchers found that with an average dose of 167 mg of the SSRI, there were no clinically significant adverse effects on cardiovascular health, specifically BP, HR, ECG indices (e.g., ST segment change), or cardiac rhythms (Wilens et al., 1999). These results agree, at least directionally, with the present investigation, which found no statistically significant changes in peripheral and central SBP, DBP, mean arterial pressure, or measures of vascular stiffness, including pulse pressure, augmentation index (AIx), or augmentation index normalized to 75 beats/min heart rate (AIx at 75). However, we may be the first to report a comprehensive assessment of blood pressure (central and peripheral) and vascular stiffness in self‐reported SSRI users versus those who were not taking SSRIs. Assessing the impacts of SSRI use on vascular health beyond the traditional brachial BP is critical as emerging indicators such as central BP and arterial stiffness are increasingly recognized as independent, and perhaps superior, indicators of CVD risk.

### 4.3 Effects of SSRIs on heart rate and heart rate variability

A quantitative review carried out by van Zyl et al. analyzed studies that evaluated the effects of SSRI treatment on HR and HRV and concluded that short‐term ECG readings demonstrate a significant decrease in HR associated with SSRIs (van Zyl et al., 2008). Additionally, van Zyl et al. found a significant increase in SDNN in SSRI users, which, along with reduced HR, would suggest greater parasympathetic nervous system activity (van Zyl et al., 2008). This differs from the present study, which found no difference in HR or SDNN with SSRIs. A 2023 meta‐analysis investigating the impact of antidepressants on the autonomic nervous system compiled 50 studies for review (Fiani et al., 2023). Study designs included randomized placebo‐controlled, crossover placebo‐controlled, prepost trials with healthy groups, and prepost trials with no control groups. Researchers found that SSRIs were associated with a significant increase in RMSSD compared to baseline measurements, indicating a potential increase in vagal efferent drive and thus greater HRV (Fiani et al., 2023). However, in the present study, there were no significant changes for other HRV measurements including LF and HF power, SDNN, PNN50, and LF/HF ratio. The present investigation only observed a significant difference in HF peak, which was lower in SSRI users, suggesting lower parasympathetic nervous system activity. Although this was the only statistically significant difference found, SSRI users had, on average, higher HR and lower values in other PNS‐mediated HRV parameters (e.g., LnRMSSD). An area of future study might include 24‐h monitoring, the effects of physical activity, or orthostatic stress on estimated cardiac autonomic activity in younger healthy individuals to get a more comprehensive assessment of potential autonomic perturbations with SSRI use.

### 4.4 Effects of SSRIs on blood glucose and lipid profile

Serodio et al. sought to determine if SSRIs and tricyclic antidepressants (TCAs) affect the relationship between obesity and CVD (Serodio et al., 2014). Using participants from the third National Health and Nutrition Examination Survey (NHANES), they discovered that SSRI users had significantly higher HDL cholesterol compared to nonusers (Serodio et al., 2014). These results differ from the present study, which revealed no difference in HDL between groups. Serodio et al. also investigated the potential effects of SSRIs on triglycerides and found no difference between users and nonusers, which aligns with the present study (Serodio et al., 2014). Furthermore, Schapir et al. investigated the metabolic effects of SSRIs by monitoring 22 adolescents (aged 8–18 years) at baseline and after 6 months of SSRI treatment and found no significant changes in glucose, cholesterol, or triglycerides (Schapir et al., 2018). However, they found that LDL trended towards elevated levels in SSRI users (Schapir et al., 2018), which is congruent with the present study. More work in the longer‐term use of SSRIs is needed to determine if there is an impact on blood glucose and lipid profile and thus CVD risk, either positive or negative, and whether this translates into higher CVD risk scores (e.g., Framingham CVD risk).

### 4.5 Effects of SSRIs on microvascular function

Research on the effects of SSRIs on vascular function is not well‐established. A 2022 systematic review by Delialis et al. analyzed five studies with a total of 323 patients, with mean ages ranging from 38 to 63 years (Delialis et al., 2022). The collected studies evaluated vascular function using the gold‐standard flow‐mediated dilation (FMD) (Delialis et al., 2022). Study designs included randomized placebo‐controlled, prospective randomized and non‐randomized clinical trials, and open‐label non‐randomized non‐interventional studies. On average, FMD increased with SSRI treatment, indicating improved conduit artery endothelial function, although microvascular function was not assessed in this meta‐analysis (Delialis et al., 2022). Additionally, Pizzi et al. investigated the effect of sertraline on inflammation and endothelial function in patients with coronary heart disease and depression (Pizzi et al., 2015). After 100 participants were randomized in a double‐blind manner, the SSRI significantly improved FMD compared to the placebo group after 20 weeks of treatment, although the reactive hyperemia or microvascular function was also not assessed in the study (Pizzi et al., 2015). The present study, using the NIRS‐VOT, a test of microvascular function, observed no significant differences in vascular function between SSRI users and nonusers. However, a large effect size of 0.9 was noted in slope 2, which was ~40% lower in the SSRI group, suggestive of lower microvascular function, and may be the first to use this technique in this application. This novel finding is worthy of further investigation. Unique to the NIRS‐VOT method, the slope 1 or deoxygenation slope, which is indicative of muscle metabolism (Greaves et al., 2024), was not different between groups and may be the first to report these assessments in this population.

### 4.6 Strengths and limitations

A major strength of this study is the matched‐pair study design. To eliminate any possible sex or participant characteristics, SSRI users were paired with nonusers according to sex, age, height, weight, and BMI. A limitation of this study is the modest sample size (*n* = 14), though using this approach with typical parameters for sample size estimation indicated an *n* = 12 would adequately power the study. Nonetheless, a larger study would be more representative of SSRI users, but providing estimates of effect size reduces the reliance on sample size and can be useful for subsequent studies. Another limitation of this study was the self‐reported and open‐label nature and not documenting how long SSRI users had been on their medication, the clinical indication for use, or dosages. Previous studies show that it may take up to 6 weeks to see the benefits of an SSRI (Harmer et al., 2009), though the participants reported being habitual users and were not naïve, and thus the data from the current study likely represent a steady state with regards to the pharmacodynamics. On the other hand, the current approach has ecological validity in that we explored the potential differences in existing SSRI users not those in a randomized controlled trial starting the drug for the first time.

## 5 CONCLUSION

Using a matched‐pairs design, the use of SSRI medication does not significantly or comprehensively affect markers of cardiometabolic health in young, healthy individuals, including BP, HR, blood glucose, and lipid profile, and thus CVD risk scores. A significantly lower HFpeak of frequency‐domain HRV was found in SSRI users, which is suggestive of lower parasympathetic activity. The present study helps contribute evidence that the use of SSRIs does not seem to produce adverse cardiovascular effects in young populations. Future studies should monitor the long‐term effects of SSRIs in young healthy adults, and perhaps any contrasts between different SSRI drugs and dosages.

## FUNDING INFORMATION

Funding was in part provided by Plexus Worldwide LLC (#2209–1044). The American Heart Association (AHA) has provided support to SJI (https://doi.org/10.58275/AHA.24AIREA1247045.pc.gr.189804).

## CONFLICT OF INTEREST STATEMENT

The authors have no conflicts of interest to disclose.

## ETHICS STATEMENT

The study was approved by the Institutional Review Board at Skidmore College (IRB#2209‐1044) and was done according to the most recent revisions to the Declaration of Helsinki.

## Data Availability

Data are available upon reasonable request to the corresponding author.
